# Supplementary material for: Substitution of polysorbates by plant-based emulsifiers: impact on vitamin D bioavailability and gut health in mice
Source: Commun Biol. 2025 Jun 7;8:896. doi: 10.1038/s42003-025-08293-4 (PMC12145454; doi:10.1038/s42003-025-08293-4)
Supplement: Supplementary file 2 — Supplemental material [file 42003_2025_8293_MOESM2_ESM.pdf]

Supplementary Material

Supplementary Table 1. List of mice primers used for RT-qPCR experiments

Name	Forward (5'→3')	Reverse (5'→3')
TNF-α	CATCTTCTCAAAATTCGAGTGACAA	TGGGAGTAGACAAGGTACAACCC
IL-6	ACAAGTCGGAGGCTTAATTACACAT	TTGCCATTGCACAACTCTTTTC
CXCL-1	CATCCGCTATCAGCTAAACCA	CAGAAGCGTCTGTGCTGTGT
Reg3-γ	ATGACCCGACACTGGGCTAT	AGAGGAAGGATTCGTCTCCCA
Muc2	GTCCGAAGTGTTACCCTGGAAT	CAGCTCTCGATGTGTGTGTAGGT
Muc3	TGGTCAACTGCGAGAATGGA	TACGCTCTCCACCAGTTCCT
Meprin-β	TTGCCAGCTCCGGAAAAGTT	AAGGTCCAGACCCAAACCTTG
Klf4	CTCCCGTCCTTCTCCACGTT	TCCTCACGCCAACGGTTAGT

Supplementary Note 1. Exposition to emulsifiers

A wide range of products contains different emulsifiers (more than 53.8% of industrial food products (including beverages) include at least one emulsifier [1,2]). For this study, we assumed as "plausible" a situation where an adult of 70 kg consumes as 10% of his diet an emulsion-based product (200mL out of 2 kg of food) with 10% of oil stabilized with a solution containing either 2% of T80, 4% of PPI or 4% PPI+ 0.9% CAX. These levels of emulsifiers fall into normal levels used by pharma and food industries. For the mice experiments, we therefore substituted 10% of the diet by this emulsion, which resulted in an exposition of 180 mg/kg body weight/day for T80, 360 mg/kg body weight/day for PPI, and 441 mg/kg body weight/day for PPI+CAX. In this scenario, exposition to T80 when converted to human according to Reagan-Shaw et al. [3]- with their equation that takes into consideration several parameters of biology, including oxygen utilization, caloric expenditure, basal metabolism, blood volume, circulating plasma proteins, and renal function-, is of 14.59 mg/kg body weight/day. Values are below the acceptable daily intake (ADI) reported by the European Food Safety Agency (EFSA) (25 mg/kg body weight/day) [4]. For pea proteins and arabinoxylans, no ADI exists as they are molecules widely present in our diet at much higher doses.

Daily exposure to emulsifiers for a standard mouse of 20 g was of 3.6 mg T80, 7.2 mg PPI or 7.2 mg PPI and 1.62 mg CAX.

<u>Calculations for T80 exposure</u>

$$\boldsymbol{Mice\ exposition} = \frac{200\ \mu\text{L emulsion}}{\text{mice}} * \frac{90\ \mu\text{L emulsifying solution}}{100\ \mu\text{L emulsion}} * \frac{2\ \text{mg T80}}{100\ \mu\text{L emulsifying solution}} * \frac{1\ \text{mice}}{20\ \text{g weight}}$$

$$= 0.18\frac{\text{mg T80}}{\text{g weight}} = 180\frac{\text{mg T80}}{\text{kg weight}}$$

$$\boldsymbol{Human\ exposition\ equivalence}^{3} = 180\frac{\text{mg T80}}{\text{kg weight mice}} * \frac{3}{37} = 14.59\frac{mg\ T80}{Kg\ weight\ humans}$$

<u>Calculations for PPI exposure</u>

$$\boldsymbol{Mice\ exposition} = \frac{200\ \mu\text{L emulsion}}{\text{mice}} * \frac{90\ \mu\text{L emulsifying solution}}{100\ \mu\text{L emulsion}} * \frac{4\ \text{mg PPI}}{100\ \mu\text{L emulsifying solution}} * \frac{1\ \text{mice}}{20\ \text{g weight}}$$

$$= 0.36\frac{\text{mg PPI}}{\text{g weight}} = 360\frac{\text{mg PPI}}{\text{kg weight}}$$

$$\boldsymbol{Human\ exposition\ equivalence}^{3} = 360\frac{\text{mg PPI}}{\text{kg weight mice}} * \frac{3}{37} = 29.19\frac{mg\ PPI}{Kg\ weight\ humans}$$

Calculations for PPI+CAX exposure

$$\textbf{\textit{Mice exposition}} = \frac{200\ \mu\text{L emulsion}}{\text{mice}} * \frac{90\ \mu\text{L emulsifying solution}}{100\ \mu\text{L emulsion}} * \frac{4\text{ mg PPI} + 0.9\text{ mg CAX}}{100\ \mu\text{L emulsifying solution}} * \frac{1\text{ mice}}{20\text{ g weight}}$$
$$= 0.441\,\frac{\text{mg PPI} + \text{CAX}}{\text{g weight}} = 441\ \frac{\text{mg PPI} + \text{CAX}}{\text{kg weight}}$$

$$\textbf{\textit{Human exposition equivalence}}^{3} = 441\,\frac{\text{mg PPI+CAX}}{\text{kg weight mice}} * \frac{3}{37} = 35.76\ \frac{mg\ \text{PPI+CAX}}{Kg\ weight\ humans}$$

Supplementary references

1. Chazelas, E. *et al.* Food additives: distribution and co-occurrence in 126,000 food products of the French market. *Sci. Rep.* **10**, 3980 (2020).
2. Open Food Facts. https://world.openfoodfacts.org/discover (2024).
3. Reagan-Shaw, S., Nihal, M. & Ahmad, N. Dose translation from animal to human studies revisited. *FASEB J.* **22**, 659–661 (2008).
4. EFSA Panel on Food Additives and Nutrient Sources added to Food (ANS). Scientific Opinion on the re-evaluation of polyoxyethylene sorbitan monolaurate (E 432), polyoxyethylene sorbitan monooleate (E 433), polyoxyethylene sorbitan monopalmitate (E 434), polyoxyethylene sorbitan monostearate (E 435) and polyoxyethylene sorbitan tristearate (E 436) as food additives. *EFSA J.* **13**, (2015).

Supplementary Figure 1

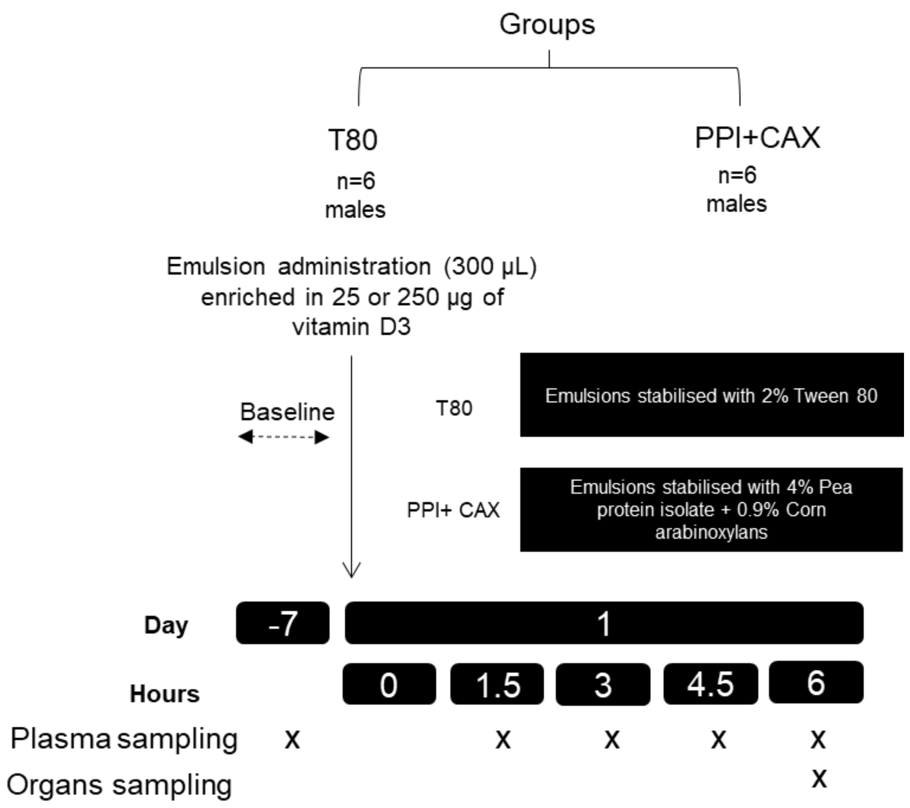

Figure S1. Experimental design for postprandial experiments.

See associated Figure 1.

Supplementary Figure 2

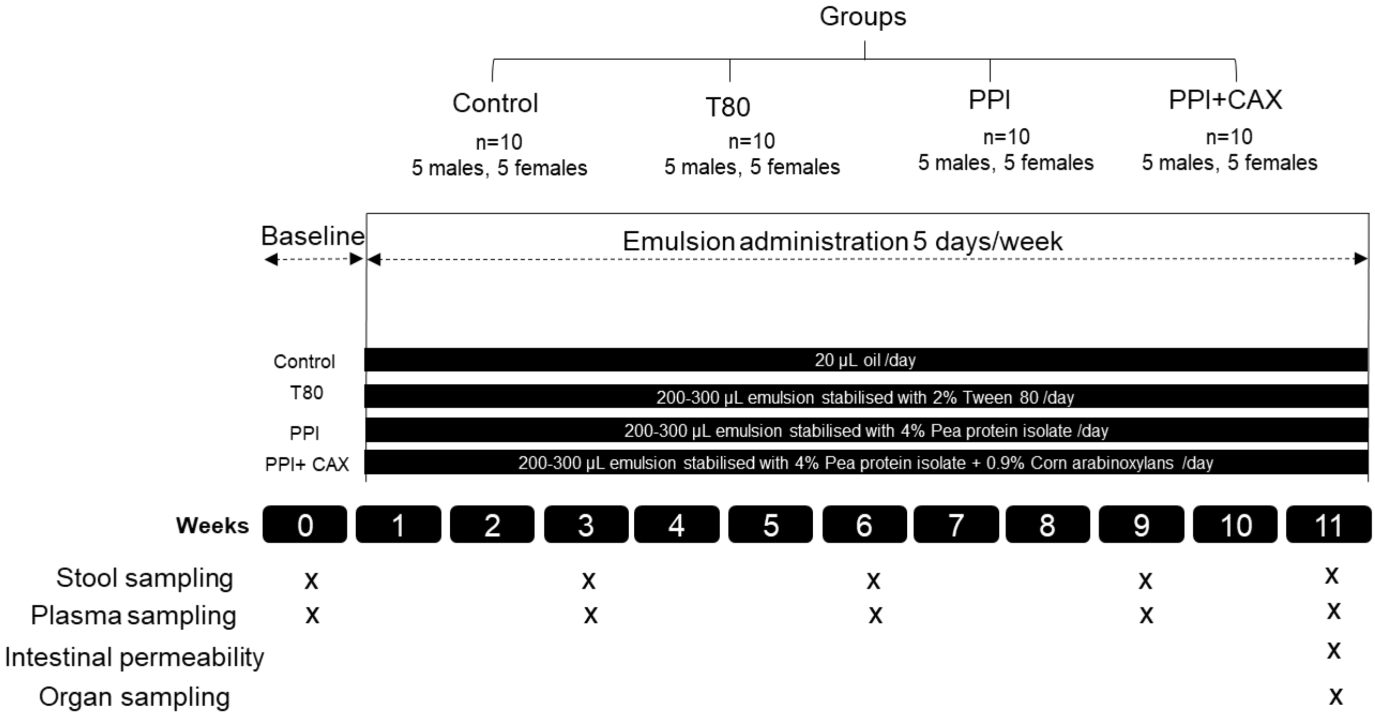

Figure S2. Experimental design for *in vivo* chronic exposure intervention.

See associated Figures 2-6.

Supplementary Figure 3

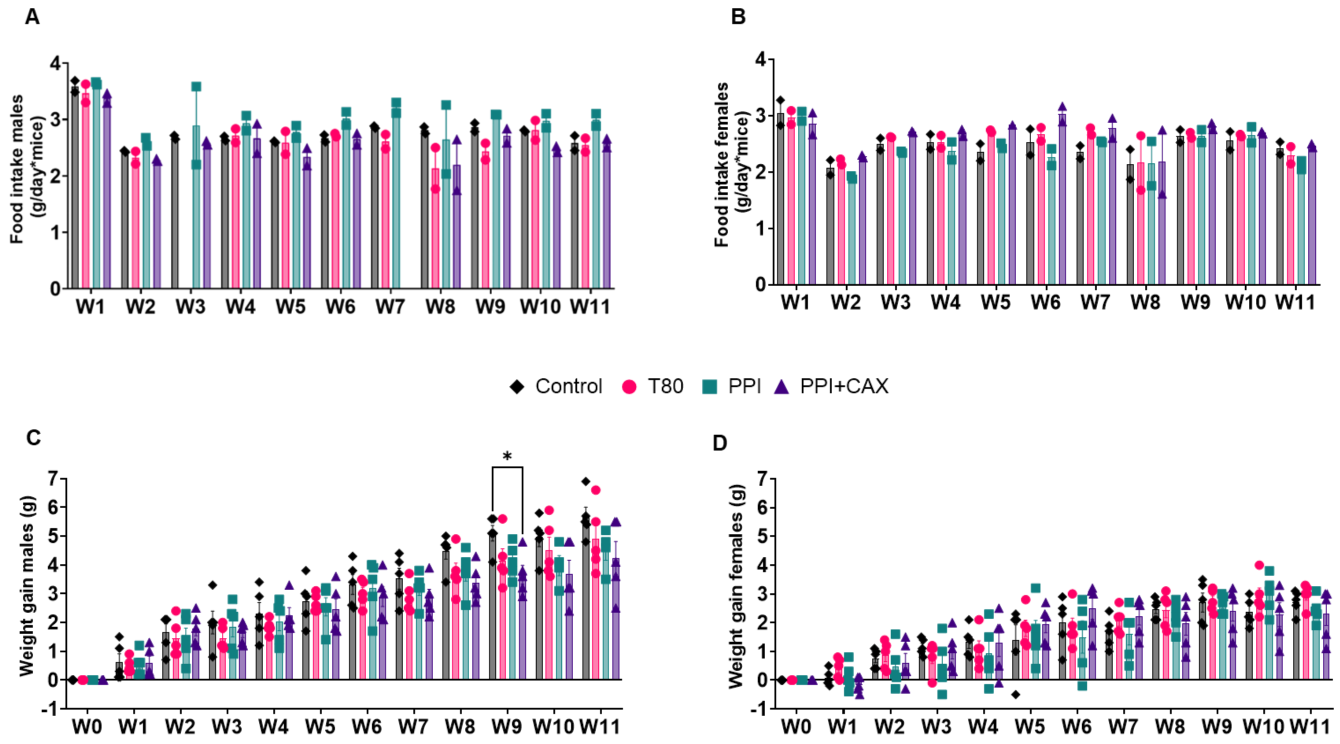

Figure S3. Mice weight gain and food intake during the chronic exposure intervention.

Control group (Control, diamonds), 2% Tween80 group (T80, circles), 4%Pea protein isolate (PPI, squares), 4%PPI+0.9%Corn arabinoxylans (PPI+CAX, triangles).

Food intake of A) Female and B) Male Weight gain of C) Female and B) Male mice from week 0 (W0) to week 11 (W11).

All graphic values are represented as mean ± SEM with n=10 mice/group including 5 males and 5 females. An asterisk indicates significant differences between groups (* $p<0.05$).

Supplementary Figure 4

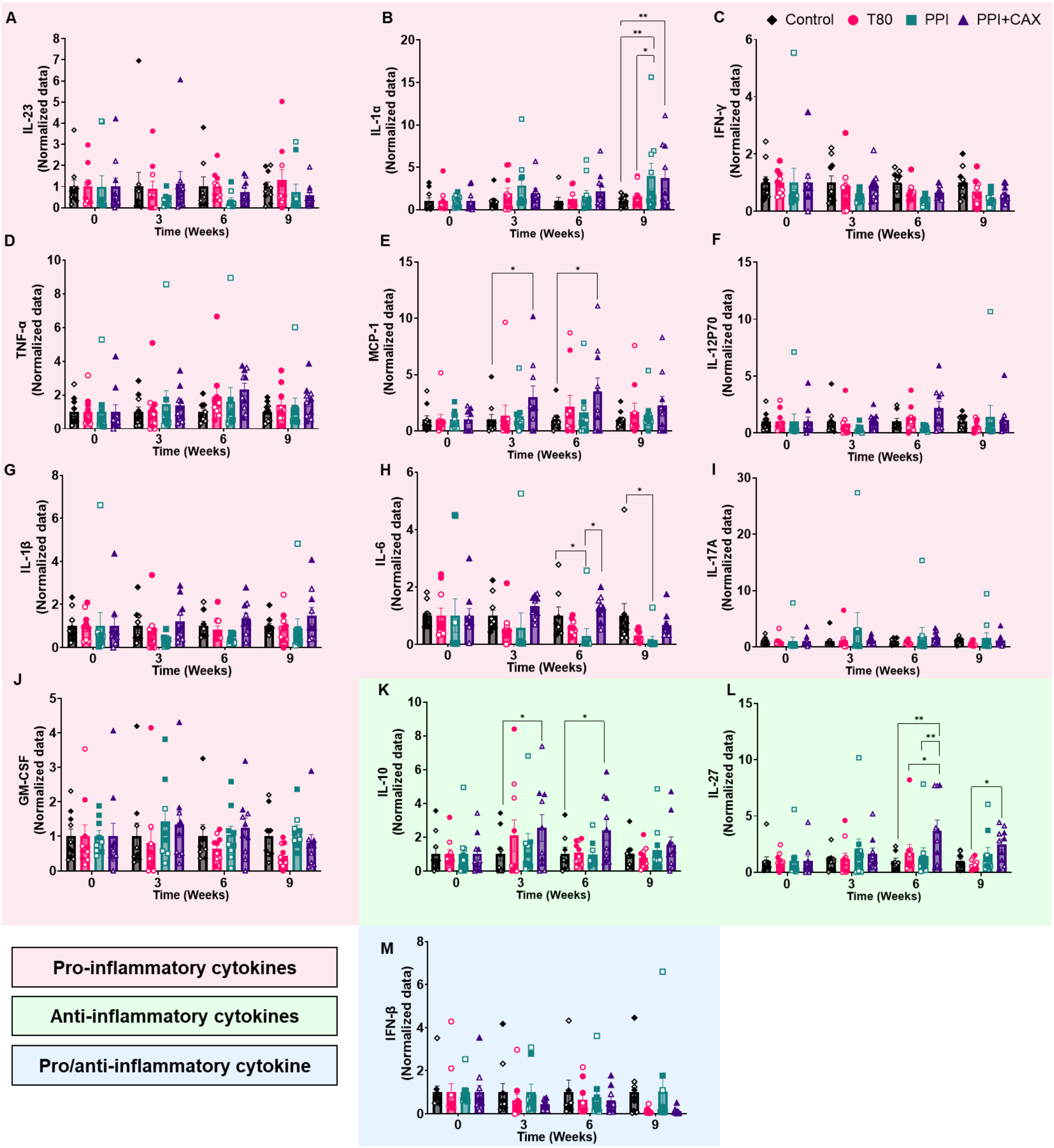

Figure S4. Plasma cytokine profiles after chronic exposure of mice to emulsifier-stabilized emulsions at dietary doses.

Normalized levels of plasmatic cytokines compared to control group including both males and females from weeks 0 to 11. Control group (Control, diamonds), 2% Tween80 group (T80, circles), 4% Pea protein isolate (PPI, squares), 4%PPI+0.9%Corn arabinoxylans (PPI+CAX, triangles). Males are represented by full symbols and females by empty symbols.

All graphic values are represented as mean ± SEM with n=10 mice/group including 5 males and 5 females. An asterisk indicates significant differences between groups (* $p<0.05$; ** $p<0.01$).

See associated Figure 3.

Supplementary Figure 5

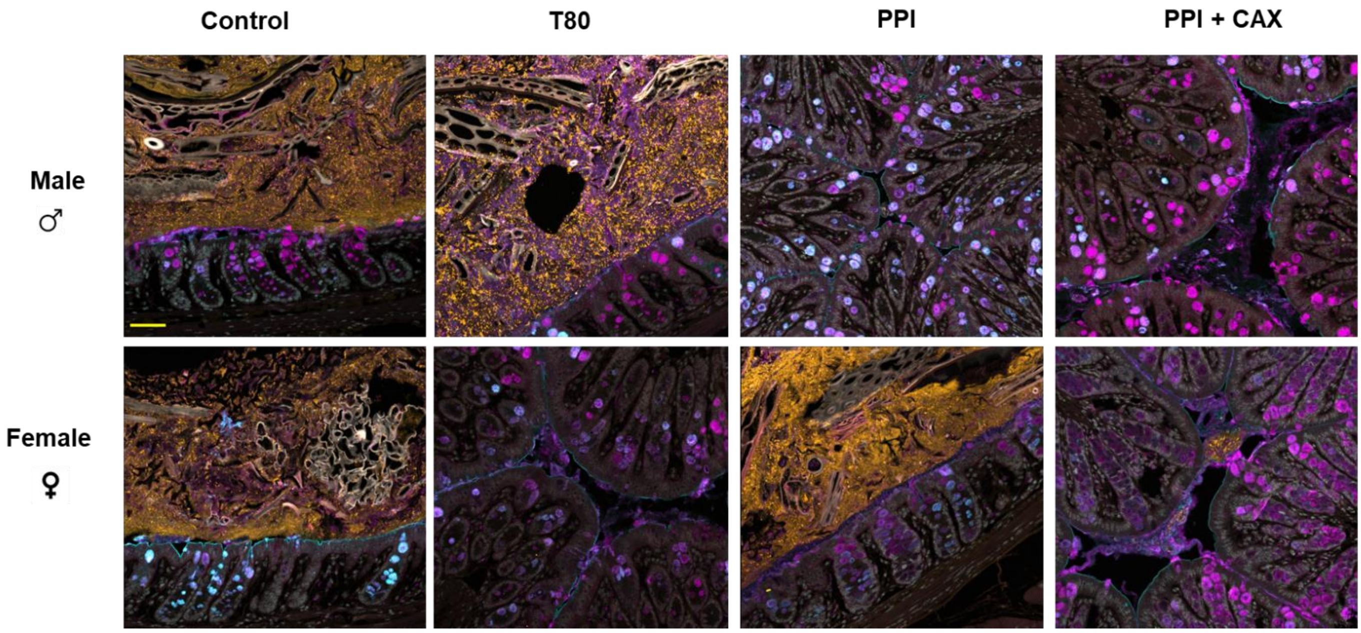

Figure S5. Colonic characteristics after chronic exposure of mice to emulsifier-stabilized emulsions at dietary doses.

Representative spectral confocal imaging projections of median jejunum from W11 mice stained by Fluorescence *in situ* Hybridization (FISH) for all bacteria (Eub-338 probe, orange), MUC2 (magenta) and Vicia Villosa Agglutinin (VVA, cyan). Mice were euthanized at fast. Bar: 50 µm.

Supplementary Figure 6

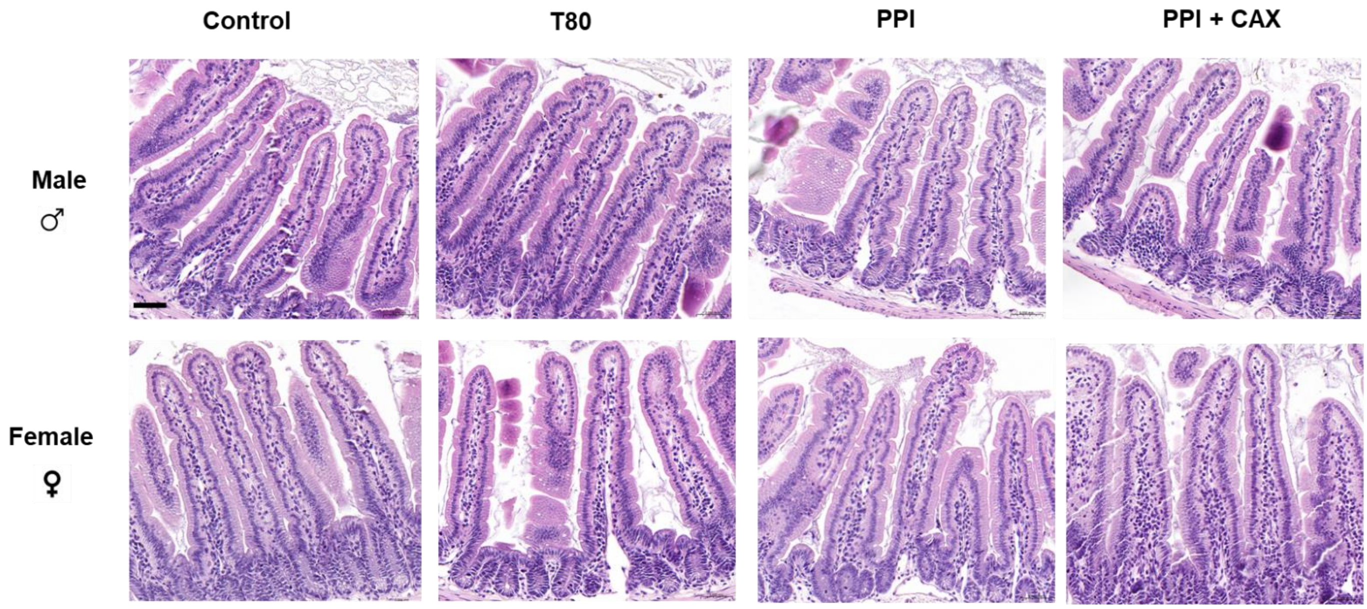

Figure S6. Jejunal characteristic and intestinal gene expression analyses related to mucosal defense systems after chronic exposure of mice to emulsifier-stabilized emulsions at dietary doses.

Representative images of median jejunum from week 11 mice stained by hematoxylin and eosin stain (HE). Mice were euthanized at fast. Bar: 50 µm.

See associated Figures 4 and 5.

Supplementary Figure 7

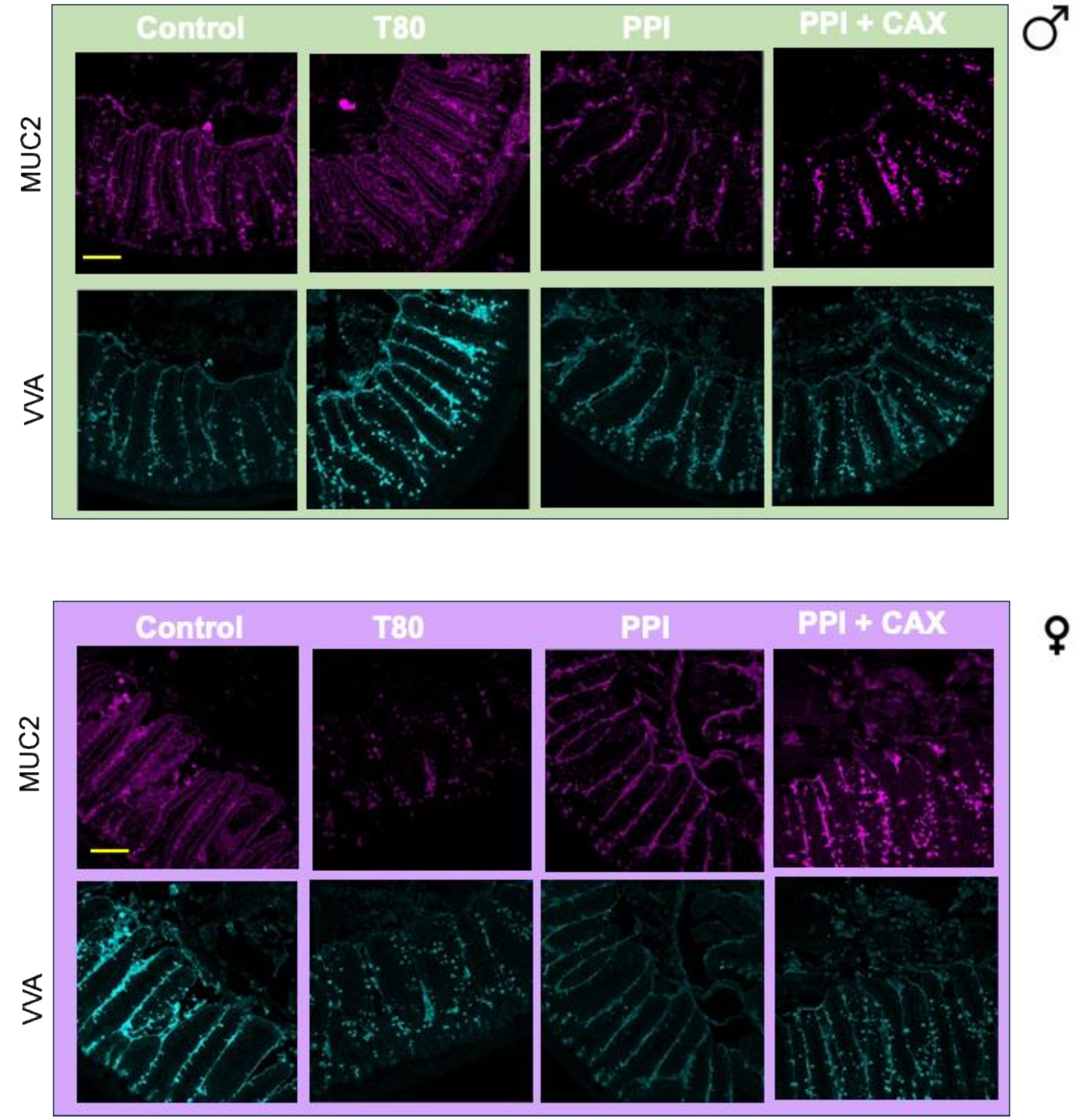

Figure S7. Jejunal characteristics of MUC2 and VVA after chronic exposure of mice to emulsifier-stabilized emulsions at dietary doses.

Representative spectral confocal imaging projections of median jejunum from week 11 mice stained by Fluorescence in situ Hybridization (FISH) for MUC2 (magenta) and Vicia Villosa Agglutinin (VVA, cyan). Mice were euthanized at fast. Bars: 50 µm.

See associated Figure 4.

Supplementary Figure 8

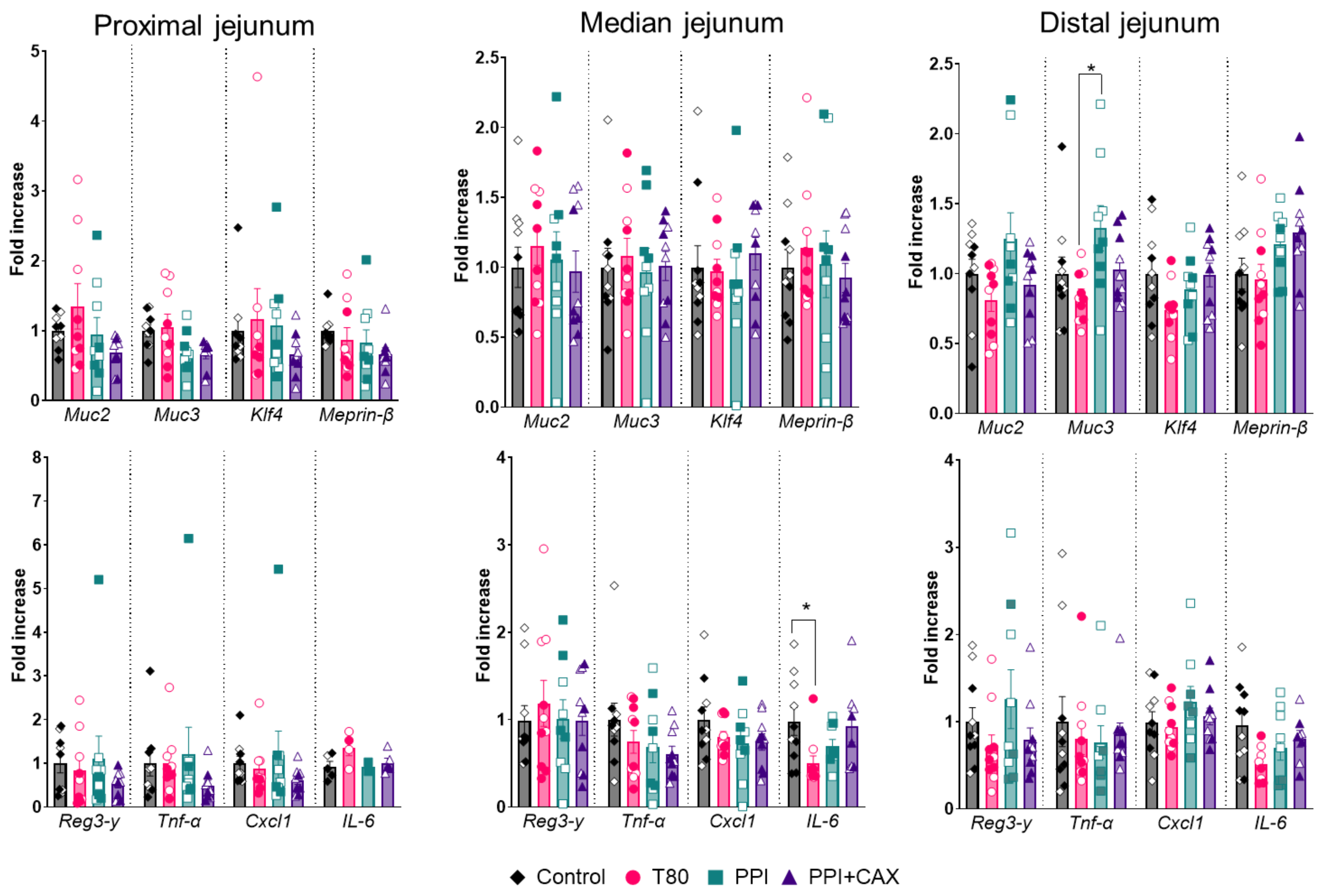

Figure S8. Intestinal gene expression analyses of selected genes related to mucosal defense.

Control group (Control, diamonds), 2% Tween80 group (T80, circles), 4% Pea protein isolate (PPI, squares), 4%PPI+0.9%Corn arabinoxylans (PPI+CAX, triangles). Mice were euthanized at fast. Males are represented by full symbols and females by empty symbols. All graphic values are represented as mean ± SEM with n=10 mice/group including 5 males and 5 females. An asterisk indicates significant differences between groups (* $p<0.05$).

See associated Figure 5.

Supplementary Figure 9

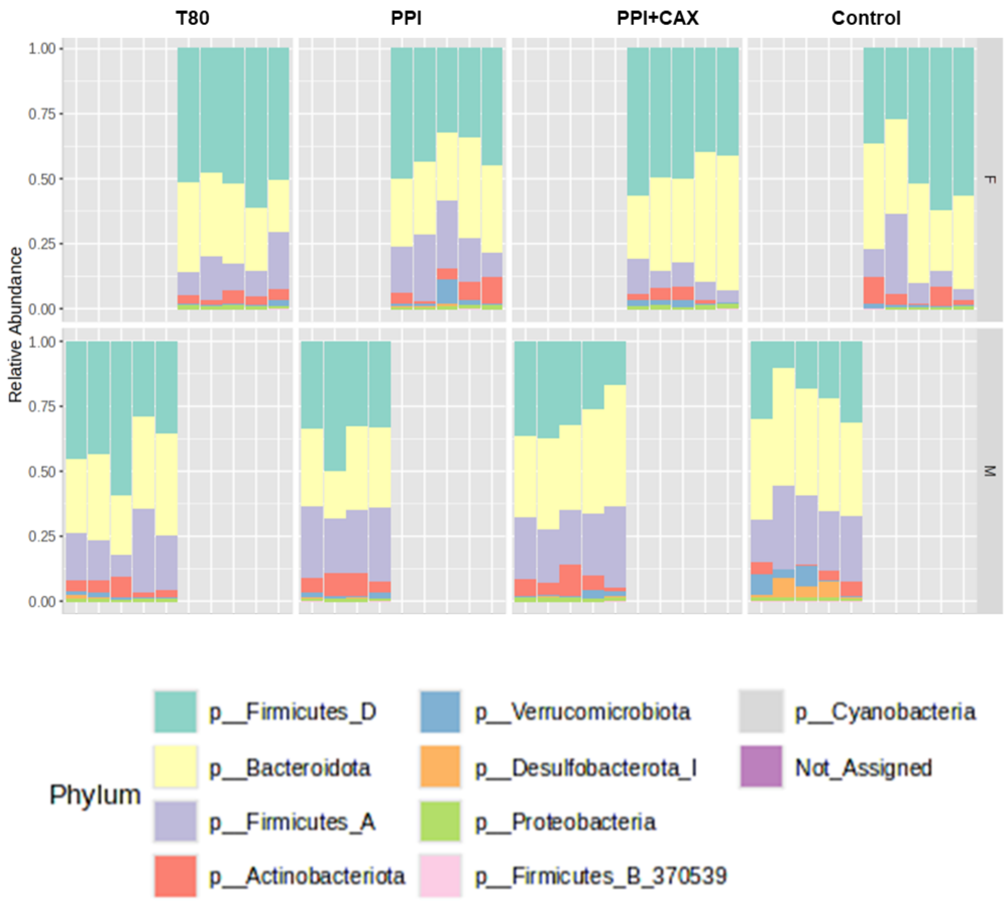

Figure S9. Individual fecal microbiota analyses after chronic exposure of mice to emulsifier-stabilized emulsions at dietary doses by sex.

Feces were collected at week 11 in ARN-free tubes.

Bar plot representation of individual composition of fecal content at the phylum level for females (top) and males (bottom).

See associated Figure 6.

Supplementary Figure 10

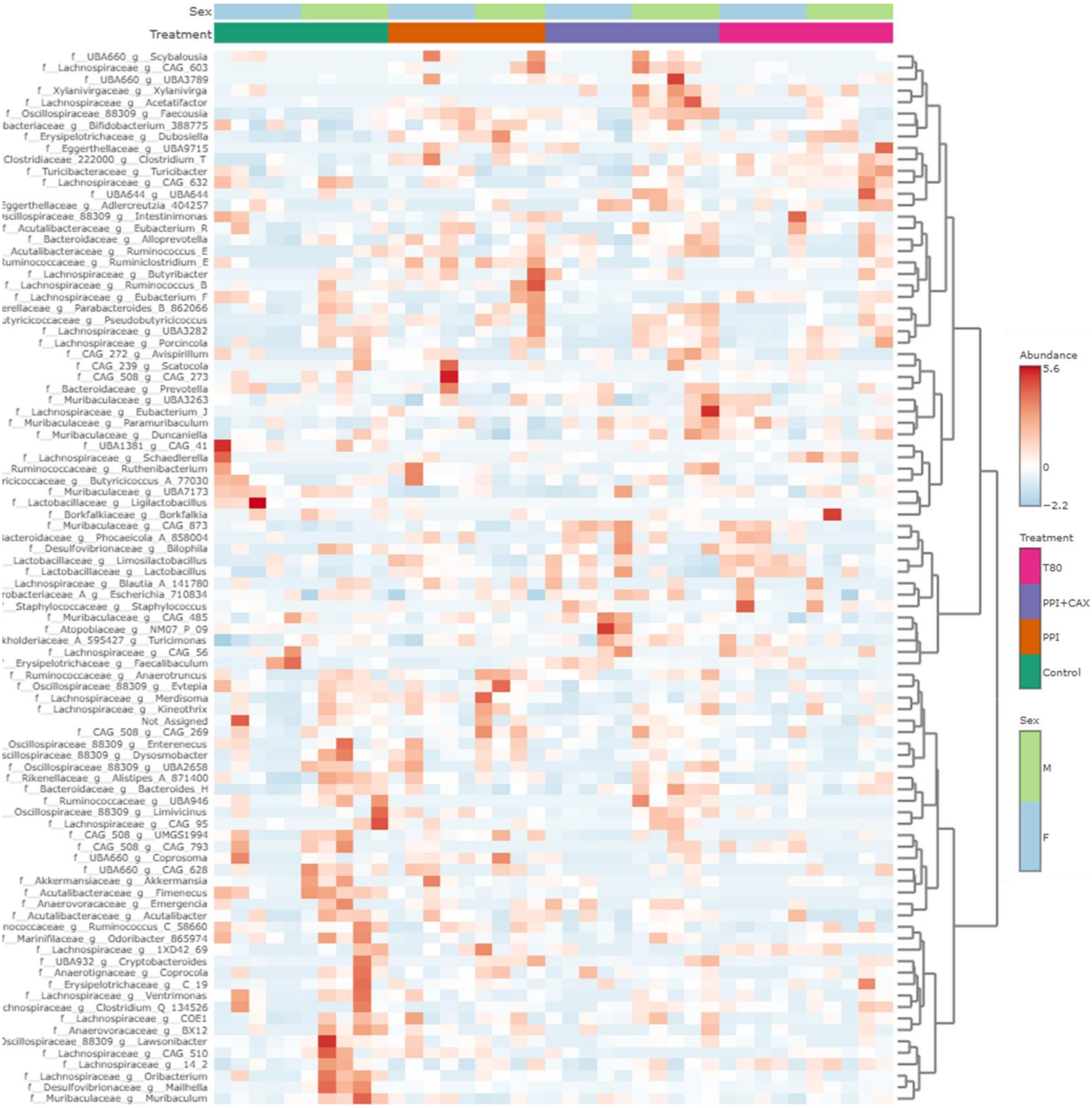

Figure S10. Heatmap of individual fecal microbiota after chronic exposure of mice to emulsifier-stabilized emulsions at dietary doses at the genus level.

Feces were collected at week 11 in ARN-free tubes
