# Supplementary material for: Substitution of polysorbates by plant-based emulsifiers: impact on vitamin D bioavailability and gut health in mice
Source: Commun Biol. 2025 Jun 7;8:896. doi: 10.1038/s42003-025-08293-4 (PMC12145454; doi:10.1038/s42003-025-08293-4)
Supplement: Supplementary file 3 — Reporting Summary [file 42003_2025_8293_MOESM3_ESM.pdf]

nature portfolio

Corresponding author(s): Emmanuelle REBOUL

Last updated by author(s): May 20, 2025

Reporting Summary

Nature Portfolio wishes to improve the reproducibility of the work that we publish. This form provides structure for consistency and transparency in reporting. For further information on Nature Portfolio policies, see our Editorial Policies and the Editorial Policy Checklist.

Statistics

For all statistical analyses, confirm that the following items are present in the figure legend, table legend, main text, or Methods section.

n/a	Confirmed	
☐	☒	The exact sample size (*n*) for each experimental group/condition, given as a discrete number and unit of measurement
☐	☒	A statement on whether measurements were taken from distinct samples or whether the same sample was measured repeatedly
☐	☒	The statistical test(s) used AND whether they are one- or two-sided<br>*Only common tests should be described solely by name; describe more complex techniques in the Methods section.*
☐	☒	A description of all covariates tested
☐	☒	A description of any assumptions or corrections, such as tests of normality and adjustment for multiple comparisons
☐	☒	A full description of the statistical parameters including central tendency (e.g. means) or other basic estimates (e.g. regression coefficient) AND variation (e.g. standard deviation) or associated estimates of uncertainty (e.g. confidence intervals)
☒	☐	For null hypothesis testing, the test statistic (e.g. *F*, *t*, *r*) with confidence intervals, effect sizes, degrees of freedom and *P* value noted<br>*Give P values as exact values whenever suitable.*
☒	☐	For Bayesian analysis, information on the choice of priors and Markov chain Monte Carlo settings
☒	☐	For hierarchical and complex designs, identification of the appropriate level for tests and full reporting of outcomes
☒	☐	Estimates of effect sizes (e.g. Cohen's *d*, Pearson's *r*), indicating how they were calculated

Our web collection on statistics for biologists contains articles on many of the points above.

Software and code

Policy information about availability of computer code

Data collection	does not apply
Data analysis	does not apply

For manuscripts utilizing custom algorithms or software that are central to the research but not yet described in published literature, software must be made available to editors and reviewers. We strongly encourage code deposition in a community repository (e.g. GitHub). See the Nature Portfolio guidelines for submitting code & software for further information.

Data

Policy information about availability of data

All manuscripts must include a data availability statement. This statement should provide the following information, where applicable:

- Accession codes, unique identifiers, or web links for publicly available datasets
- A description of any restrictions on data availability
- For clinical datasets or third party data, please ensure that the statement adheres to our policy

Data availabe at https://doi.org/10.57745/AFURVW.
Unprocessed sequencing data are deposited in the European Nucleotide Archive under accession number PRJEB89673

Research involving human participants, their data, or biological material

Policy information about studies with human participants or human data. See also policy information about sex, gender (identity/presentation), and sexual orientation and race, ethnicity and racism.

Reporting on sex and gender	does not apply
Reporting on race, ethnicity, or other socially relevant groupings	does not apply
Population characteristics	does not apply
Recruitment	does not apply
Ethics oversight	does not apply

Note that full information on the approval of the study protocol must also be provided in the manuscript.

Field-specific reporting

Please select the one below that is the best fit for your research. If you are not sure, read the appropriate sections before making your selection.

☒ Life sciences ☐ Behavioural & social sciences ☐ Ecological, evolutionary & environmental sciences

For a reference copy of the document with all sections, see nature.com/documents/nr-reporting-summary-flat.pdf

Life sciences study design

All studies must disclose on these points even when the disclosure is negative.

Sample size	n=10, 5 male, 5 female
Data exclusions	No data excluded unless experimental error
Replication	Not replicated
Randomization	Mice were randomly placed in the different groups
Blinding	Qualitative analysis of images was blinded (the person doing image analysis did not know the group or sex of the mice), quantitative analysis was not blinded

Reporting for specific materials, systems and methods

We require information from authors about some types of materials, experimental systems and methods used in many studies. Here, indicate whether each material, system or method listed is relevant to your study. If you are not sure if a list item applies to your research, read the appropriate section before selecting a response.

Materials & experimental systems

n/a	Involved in the study	
☐	☒	Antibodies
☐	☒	Eukaryotic cell lines
☒	☐	Palaeontology and archaeology
☐	☒	Animals and other organisms
☒	☐	Clinical data
☒	☐	Dual use research of concern
☒	☐	Plants

Methods

n/a	Involved in the study	
☒	☐	ChIP-seq
☐	☒	Flow cytometry
☒	☐	MRI-based neuroimaging

Antibodies

Antibodies used	Mouse inflamation Panel Detection Antibodies from the kit LEGENDplex Mouse Inflammation Panel (13-plex) with V-botton Plate, biolegend, (Catalog number 740446)<br>Anti-mouse IgG Horseradish Peroxidase linked (CliniScience, 01017-05)

	Goat anti-mouse IgG Fab-HRP (Southern Biotech, Cliniscience 1015-05)<br>anti-MUC2, Santa Cruz Biotechnology, Cat#sc-15334; AB_2146667<br>anti-VVA, Vector Labs, B-1235-2<br>Mouse antibodies from the kit Mouse Lipocalin-2/NGAL DuoSet ELISA, Bio-Techne SAS, Catalog #: DY1857
Validation	Mouse inflamation Panel Detection Antibodies :Validated by the manufacturer<br>Anti-mouse IgG Horseradish Peroxidase linked : Validated by manufacturer<br>Goat anti-mouse IgG Fab-HRP : Validated by manufacturer<br>anti-MUC2 and anti-VVA validated by manufacturer and in the lab for specific conditions<br>Mouse antibodies from the kit Mouse Lipocalin-2/NGAL DuoSet ELISA, validated by manufacturer

Eukaryotic cell lines

Policy information about cell lines and Sex and Gender in Research

Cell line source(s)	Caco2-TC7, HEK-TLR4 and HEK-TLR5
Authentication	For Caco2-TC7 Morphology check by microscope, growth curve analysis, control number of passages<br>For HEK-TLR4 and HEK-TLR5 Indirect authentication through the use of selection agents and the creation of a standard range to validate TLR4 or TLR5 expression.
Mycoplasma contamination	Tested negative for mycoplasma contamination for all lines
Commonly misidentified lines<br>(See ICLAC register)	does not apply

Animals and other research organisms

Policy information about studies involving animals; ARRIVE guidelines recommended for reporting animal research, and Sex and Gender in Research

Laboratory animals	Wild-type Mice C57BL/6 aged 6-7 weeks
Wild animals	does not apply
Reporting on sex	Only male mice were used for postpandrial evaluation of vitamin uptake<br>Sex was considered for the experimental design and analyses for inflammation experiments
Field-collected samples	does not apply
Ethics oversight	The used protocols were approved by the "Ministère de l'Education Nationale, de l'Enseignement Supérieur, et de la Recherche" of France (approval numbers APAFIS#13473-2018020918403330v3 and APAFIS#46024-2023112013358971v4 for postprandial experiments and APAFIS#40225-202301031635597v4 for the chronic exposure experiment, respectively).

Note that full information on the approval of the study protocol must also be provided in the manuscript.

Plants

Seed stocks	does not apply
Novel plant genotypes	does not apply
Authentication	does not apply

Flow Cytometry

Plots

Confirm that:

☐ The axis labels state the marker and fluorochrome used (e.g. CD4-FITC).

☐ The axis scales are clearly visible. Include numbers along axes only for bottom left plot of group (a 'group' is an analysis of identical markers).

☐ All plots are contour plots with outliers or pseudocolor plots.

☐ A numerical value for number of cells or percentage (with statistics) is provided.

Methodology

Sample preparation	No cells used , flow cytometry used to quantify cytokines with the Legendplex Mouse inflamation palnel (13-plex) with v-botton plate
Instrument	Cytoflex LX
Software	LEGENDplex™ software
Cell population abundance	does not apply
Gating strategy	does not apply

☐ Tick this box to confirm that a figure exemplifying the gating strategy is provided in the Supplementary Information.
